# Epigenetic Silencing of ITGA2 by MiR-373 Promotes Cell Migration in Breast Cancer

**DOI:** 10.1371/journal.pone.0135128

**Published:** 2015-08-10

**Authors:** Wen Ding, Xiao-Lu Fan, Xuan Xu, Jin-Zhou Huang, Song-Hui Xu, Qian Geng, Rong Li, De Chen, Guang-Rong Yan

**Affiliations:** 1 Institutes of Life and Health Engineering, Jinan University, Guangzhou, China; 2 Biomedicine Research Center and Department of Surgery, The Third Affiliated Hospital of Guangzhou Medicine University, Guangzhou, China; 3 Biomedicine Research Center and Department of Surgery, The Third Affiliated Hospital of Guangzhou Medicine University, Guangzhou, China; 4 Department of Oncology, Nanfang Hospital, Southern Medical University, Guangzhou, China; 5 Key Laboratory for Major Obstetric Diseases of Guangdong Province, Key Laboratory of Reproduction and Genetics of Guangdong Higher Education Institutes, Guangzhou, China; The University of Hong Kong, CHINA

## Abstract

The loss of ITGA2 plays an important role in cancer metastasis in several solid cancers. However, the molecular mechanism of ITGA2 loss in primary cancers remains unclear. In this study, we found that a lower ITGA2 protein level was observed in breast cancers compared to adjacent non-cancerous breast tissues. Interestingly, the reduction degree of ITGA2 at the protein level was far more than that at the mRNA level. We further showed that the translation of ITGA2 mRNA was directly inhibited by miR-373 through binding to ITGA2-3’UTR. Silencing of ITGA2 detached cell-cell interactions, induced the deploymerization of stress fiber F-actin and stimulated cancer cell migration, similar to the effect of miR-373 over-expression. The co-expression of ITGA2, not ITGA2-3’UTR, could abrogate miR-373-induced cancer cell migration because that the expression of ITGA2-3’UTR was inhibited by co-transfected miR-373. ITGA2 protein level was inversely associated with miR-373 level in breast cancers (r = -0.663, P<0.001). 73.33% of breast cancer patients with high miR-373 and low ITGA2 expression exhibited the lymph node-positive metastases. Together, our results show that epigenetic silencing of ITGA2 by miR-373 stimulates breast cancer migration, and miR-373^high^/ITGA2^low^ may be as a prognosis biomarker for breast cancer patients.

## Introduction

The most critical factor in cancer survivability is whether cancer cells migrate from the primary tumor site and form distant metastases. Tumor metastasis is a complex process, comprising multiple sequential steps, such as cell-cell and cell-matrix detachment, invasion through the basement membrane, the circulation via blood vessels or lymphatic system, and location in the other tissues by cell-cell and cell-matrix interaction. In addition to dysregulated expression of matrix components in the cancer microenvironment, the loss of cell surface adhesive receptors has been found to be involved in the cancer progression and metastasis [1]. As important mediators of cell adhesion, integrins of cell surface receptors of extracellular matrix (ECM) proteins play a critical role in tumor progression and metastasis [2].

Integrin α2 (ITGA2) is an important collagen receptor on platelets and epithelial cells and is highly expressed on normal epithelium cells. Expression of ITGA2 is regulated during normal cell differentiation and is altered during tumorigenesis [3, 4]. ITGA2 knockout mice show impaired mammary gland branching morphogenesis [5]. The loss of ITGA2 in cancer cells was associated with metastatic behavior in hepatocarcinoma, colon carcinoma, and rhabdomyosarcoma [6–8]. However, the molecular mechanism of ITGA2 loss in cancer cells is still unknown.

MicroRNAs (miRNAs) are an abundant class of small non-coding RNAs, consisting of ~22 nucleotides that negatively regulate gene expression at the post-transcriptional level by blocking mRNA translation or degrading target mRNAs [9]. Some miRNAs have also been implicated in the regulation of cancer invasion and metastasis [10–14]. For example, miR-29b and miR-195 suppresses tumor angiogenesis, invasion, and metastasis [15, 16], miR-130b promotes CD133^+^ liver tumor-initiating cell growth and self-renewal [14], whereas miR-10b stimulates cancer cell invasion and metastasis [17].

In this study, we found that the reduction degree of ITGA2 protein was far more than that at the mRNA level in breast cancers. The translation of ITGA2 mRNA was directly inhibited by miR-373 by binding to ITGA2-3’UTR. The co-expression of ITGA2, not ITGA2-3’UTR, could block miR-373-induced cancer migration. ITGA2 protein level was inversely associated with miR-373 level in breast cancers. Most of breast cancer patients with miR-373^high^/ITGA2^low^ exhibited the lymph node (LN)-positive metastases.

## Results

### Down-regulation of ITGA2 protein is closely associated with cancer metastasis

**Fig 1:**
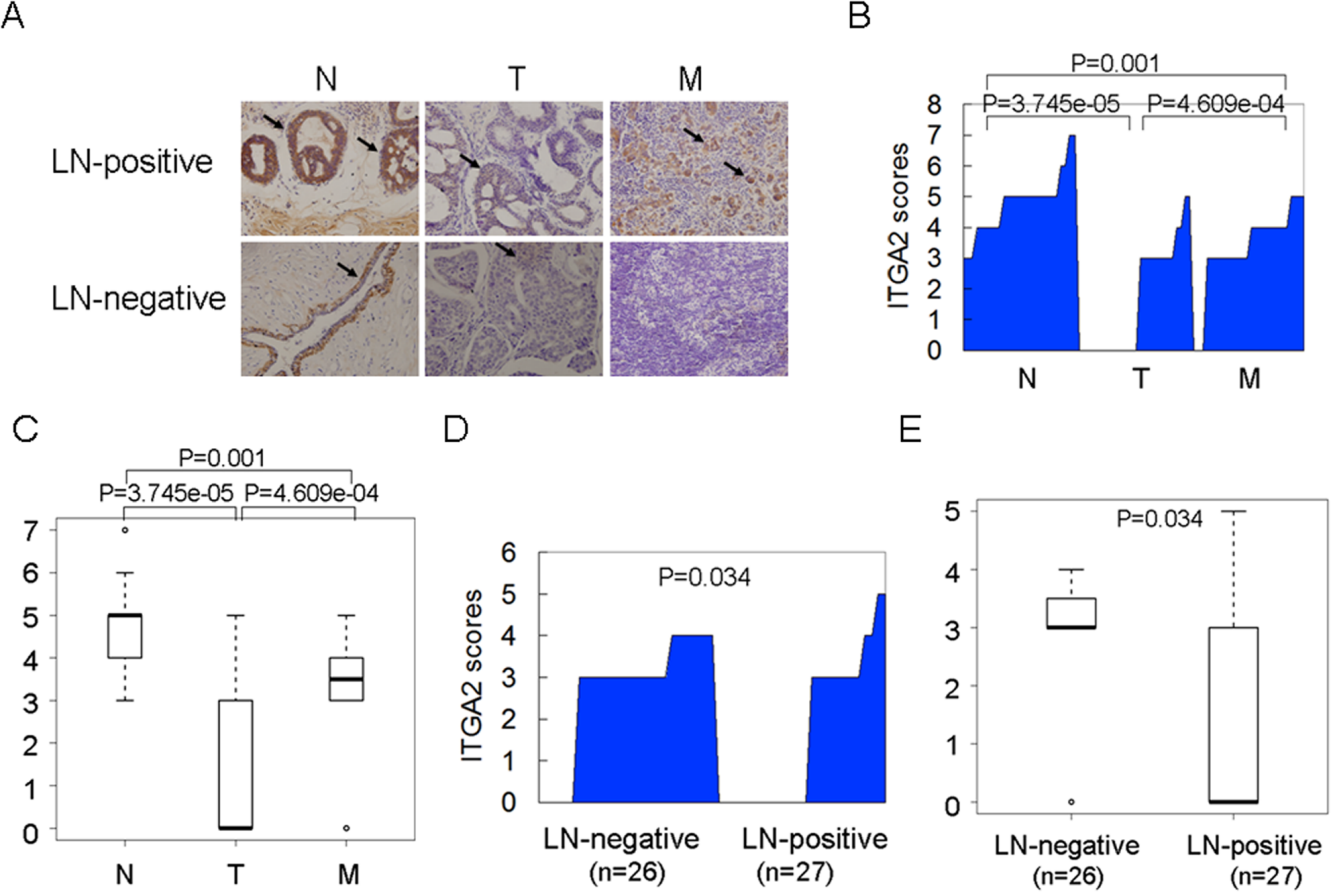
A lower ITGA2 protein level was observed in primary cancers compared to adjacent non-cancerous samples. (A) Representative images from IHC staining of ITGA2 in lymph node (LN)-positive and LN-negative breast cancers. N: adjacent non-cancerous samples; T: primary cancers; M: lymph node metastases. Images were captured at 400× magnification. (B) ITGA2 protein level was plotted in N, T and M. (C) ITGA2 protein level was shown as a box plot using IHC scores. The horizontal lines represent the median; the bottom and top of the boxes represent the 25^th^ and 75^th^ percentiles, respectively, and the vertical bars represent the range of the data. (D) ITGA2 protein level was plotted between LN-negative and positive primary breast cancers as described in (B). (E) ITGA2 protein level was shown as a box plot between LN-negative and positive primary breast cancers as described in (C).

To investigate the correlation of ITGA2 protein level with cancer metastasis phenotype, the primary breast cancers, matched adjacent non-cancerous samples and lymph node (LN) metastases from 53 human subjects (26 LN-negative and 27 LN-positive) were collected. The patient characteristics are listed in Table 1, and the representative results from the IHC analysis are shown in Fig 1A. Our data showed that ITGA2 protein level was positively associated with lymph node metastasis (P = 0.045), but was not related to TNM stage, histological grade, ER, PR, Her2, Ki-67 status, or age (Table 1).

**Table 1:** Correlations of ITGA2 expression with clinicopathological status in 53 patients with breast cancer.

	All cases	ITGA2				p Value*
		(-)	(+)	(++)	(+++)	
Age (years) 0.085						
≤ 50	36	10	18	8	0	
> 50	17	10	4	3	0	
Tumor Size (cm) 0.226						
T1 (≤ 2)	15	4	7	4	0	
T2 (2–5)	25	12	10	3	0	
T3 (> 5)	13	4	5	4	0	
TNM stage 0.195						
Ⅰ	8	2	4	2	0	
Ⅱ	20	5	10	5	0	
Ⅲ	25	13	8	4	0	
Histological Grade 0.056						
G1-2	34	9	17	8	0	
G3	19	11	5	3	0	
Lymph Node Metastasis 0.045						
N0	27	6	14	7	0	
N1	3	2	1	0	0	
N2	9	3	3	3	0	
N3	14	9	4	1	0	
ER 0.127						
Negative	15	9	3	3	0	
Positive	38	11	19	8	0	
PR 0.074						
Negative	21	12	5	4	0	
Positive	32	8	17	7	0	
HER 2 0.646						
Negative	42	17	16	9	0	
Positive	11	3	6	2	0	
Ki-67 0.703						
< 14%	9	2	6	1	0	
≥ 14%	44	18	16	10	0	

*x^2^ test.

A lower protein level of ITGA2 was detected in primary breast cancers relative to adjacent non-cancerous breast tissues (P<0.001); however, ITGA2 was recovered in lymph node metastases compared to the primary cancers (P<0.001) (Fig 1B and 1C). And ITGA2 protein level was lower in patients with LN-positive metastases compared to LN-negative metastases (P = 0.034) (Fig 1D and 1E). Taken together, our observations revealed that ITGA2 protein level is significantly reduced in breast cancers, especially in breast cancers with the LN-positive metastases, and its reduction might play an important role in cancer invasion and metastasis.

### MiR-373 directly suppresses ITGA2 level by translation inhibition

**Fig 2:**
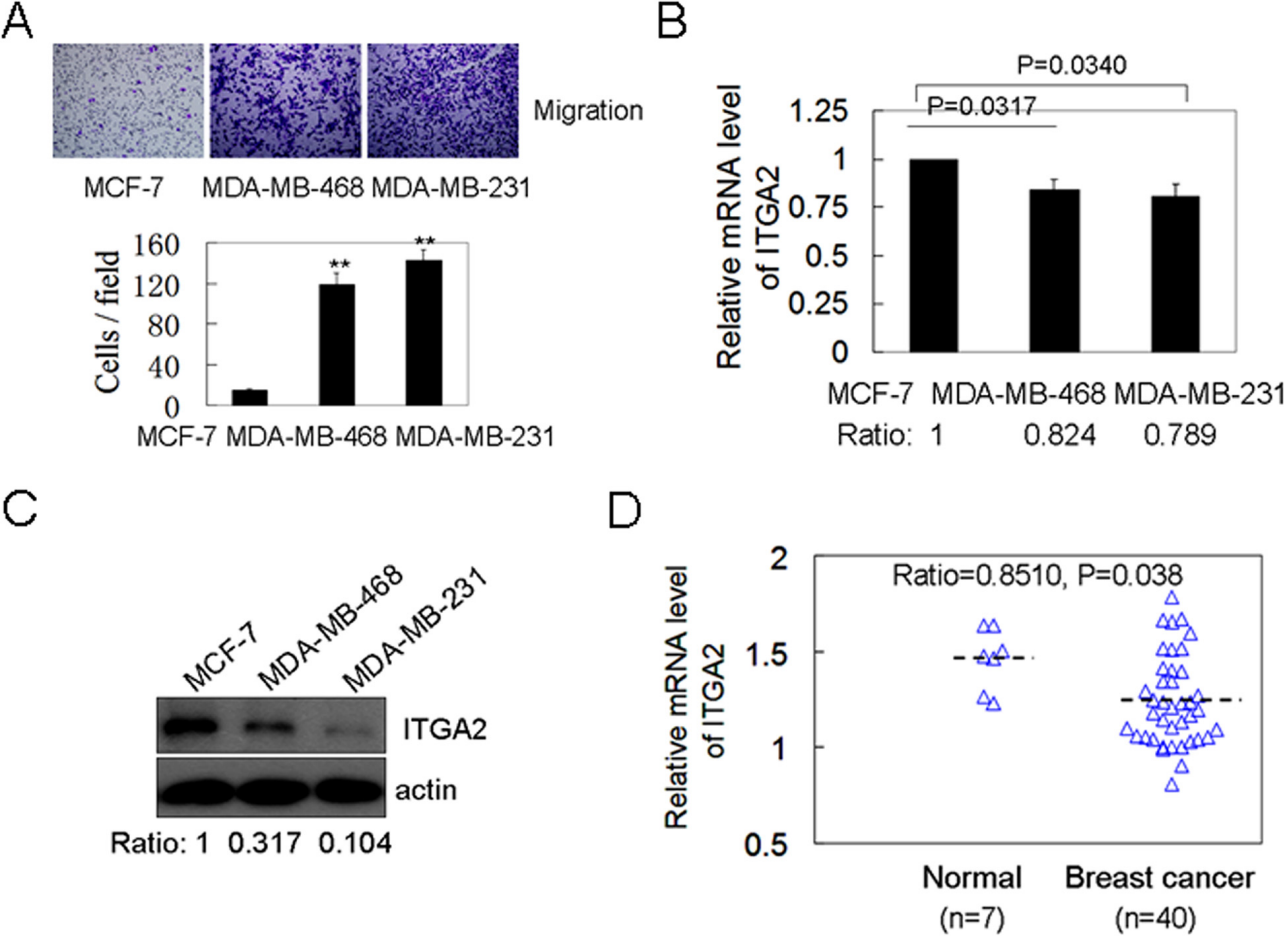
The reduction degree of ITGA2 protein was more than that at the mRNA level. (A) The migration abilities at the indicated breast cancer cell lines were detected. (B) The mRNA level of ITGA2 at the indicated breast cancer cell lines was analyzed by qRT-PCR. (C) The ITGA2 protein level at the indicated breast cancer cell lines was determined by western blotting. (D) The mRNA levels of ITGA2 in the publicly available microarray data from the Richardson cohort were plotted.

To investigate the molecular mechanism of ITGA2 loss in cancers, the mRNA level of ITGA2 was further analyzed. We found that the mRNA level of ITGA2 in high-metastatic cells MDA-MB-468 and MDA-MB-231 was slightly decreased compared to the non-metastatic breast cell MCF-7 (P = 0.0317 and 0.0340) (Fig 2A and 2B). However, the reduction degree of ITGA2 protein was more than that at the mRNA level, as shown in Fig 2C. The publicly accessible microarray data, organized by Richardson, was further analyzed, including 40 primary breast cancers and 7 normal breast tissues [18]. We also found that the mRNA level of ITGA2 was slightly reduced in breast cancers versus normal breast tissues (P = 0.038) (Fig 2D). These observations showed that the ITGA2 down-regulation in breast cancers is mainly mediated at the post-transcriptional level.

**Fig 3:**
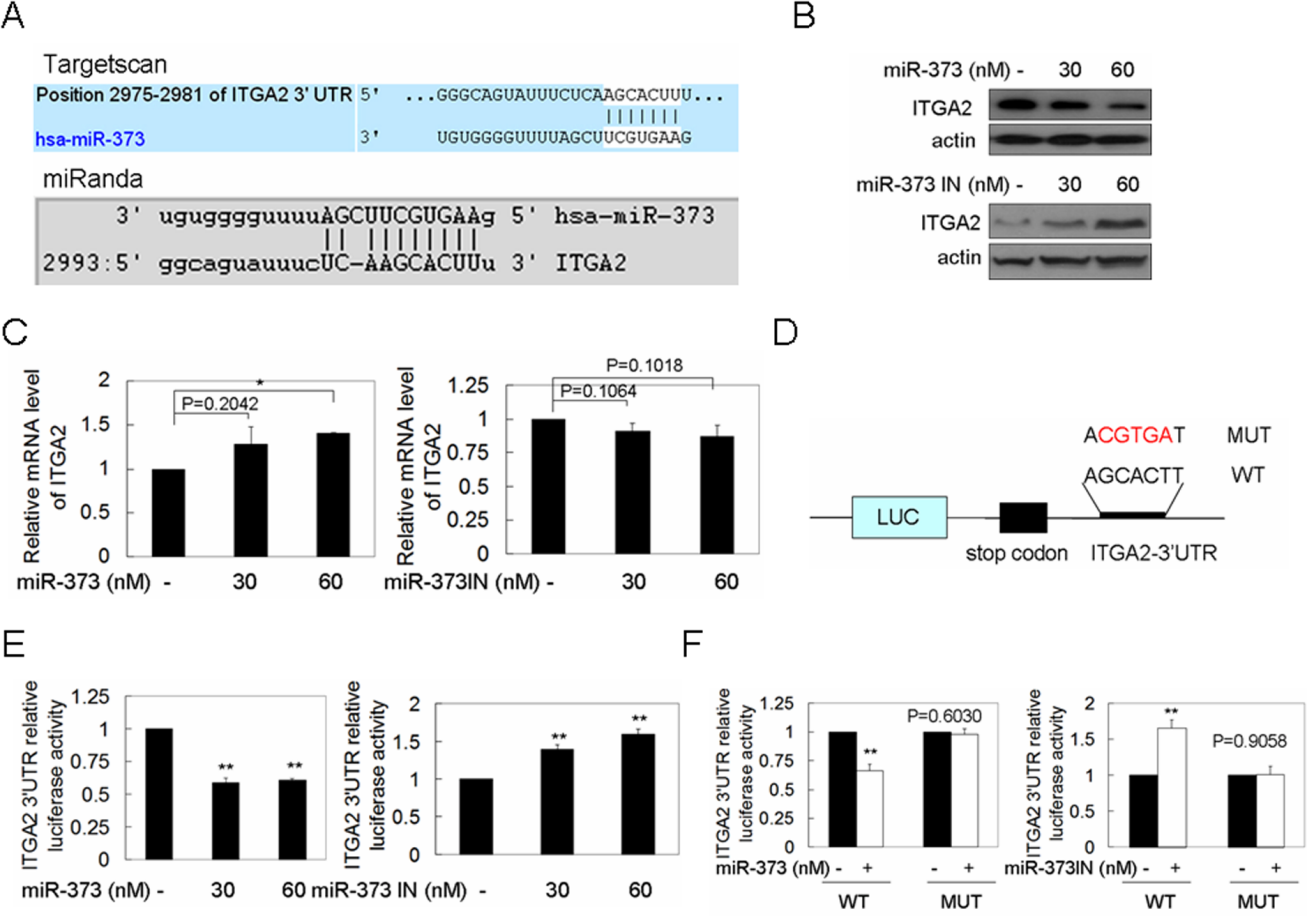
The translation of ITGA2 mRNA was directly inhibited by miR-373 by binding to its 3’UTR. (A) The binding site of miR-373 in 3’UTR of ITGA2 was simultaneously predicted by TargetScan and miRanda program. (B) MCF-7 and MDA-MB-231 cells were transfected with miR-373 precursor (upper panel) and inhibitor (miR-373 IN) (low panel) at the indicated concentration, respectively, ITGA2 protein level was analyzed by western blot. (C) The cells were treated as described in (A), and then the mRNA level of ITGA2 was analyzed by qRT-PCR (n = 3). (D) Schematic diagram of the reporter constructs of 3’UTR of ITGA2 of wild type (WT) and mutation type (MUT). (E) MCF-7 or MDA-MB-231 cells were co-transfected with miR-373 precursor or inhibitor at the indicated concentration together with the 3’UTR of ITGA2 reporter plasmid, respectively, and then luciferase activity was measured using the dual-luciferase reporter assay system (n = 3). (F) MCF-7 or MDA-MB-231 cells were co-transfected with miR-373 precursor or inhibitor together with the indicated reporter plasmid, respectively, and then luciferase activity was measured as described in (E) (*p<0.05 and **p<0.01).

MiRNAs negatively regulate the expression of target genes mainly through translation inhibition in mammal cells. MiRNAs which might bind to the 3’UTR sequences of IGTA2 were analyzed by Targetscan and miRanda program. A binding site of miR-373 was found in the 3’UTR region of ITGA2 (Fig 3A). We further demonstrated that miR-373 over-expression down-regulated the ITGA2 protein level, while miR-373 inhibition increased the ITGA2 protein level in a dose-dependent manner (Fig 3B). However, the mRNA level of ITGA2 was not changed by miR-373 (Fig 3C), suggesting that miR-373 regulates ITGA2 protein level at translation level, not induction of mRNA degradation.

To investigate whether miR-373 directly suppresses ITGA2 level, the luciferase reporter constructs containing ITGA2-3’UTR were co-transfected with miR-373 precursor into MCF-7 cells (Fig 3D). MiR-373 overexpression decreased the luciferase activity, whereas the luciferase activity was not changed when the 3’UTR sequences in the complementary sites for the seed region of miR-373 were mutated (Fig 3E and 3F). Furthermore, the luciferase activity was significantly increased by miR-373 inhibition (Fig 3E), whereas the miR-373 inhibition did not change the luciferase activity when the binding sites were mutated (Fig 3F). Altogether, these data reveal that miR-373 directly suppresses the translation of ITGA2 by binding to the ITGA2-3’UTR.

### Silencing of ITGA2 induces cancer migration

**Fig 4:**
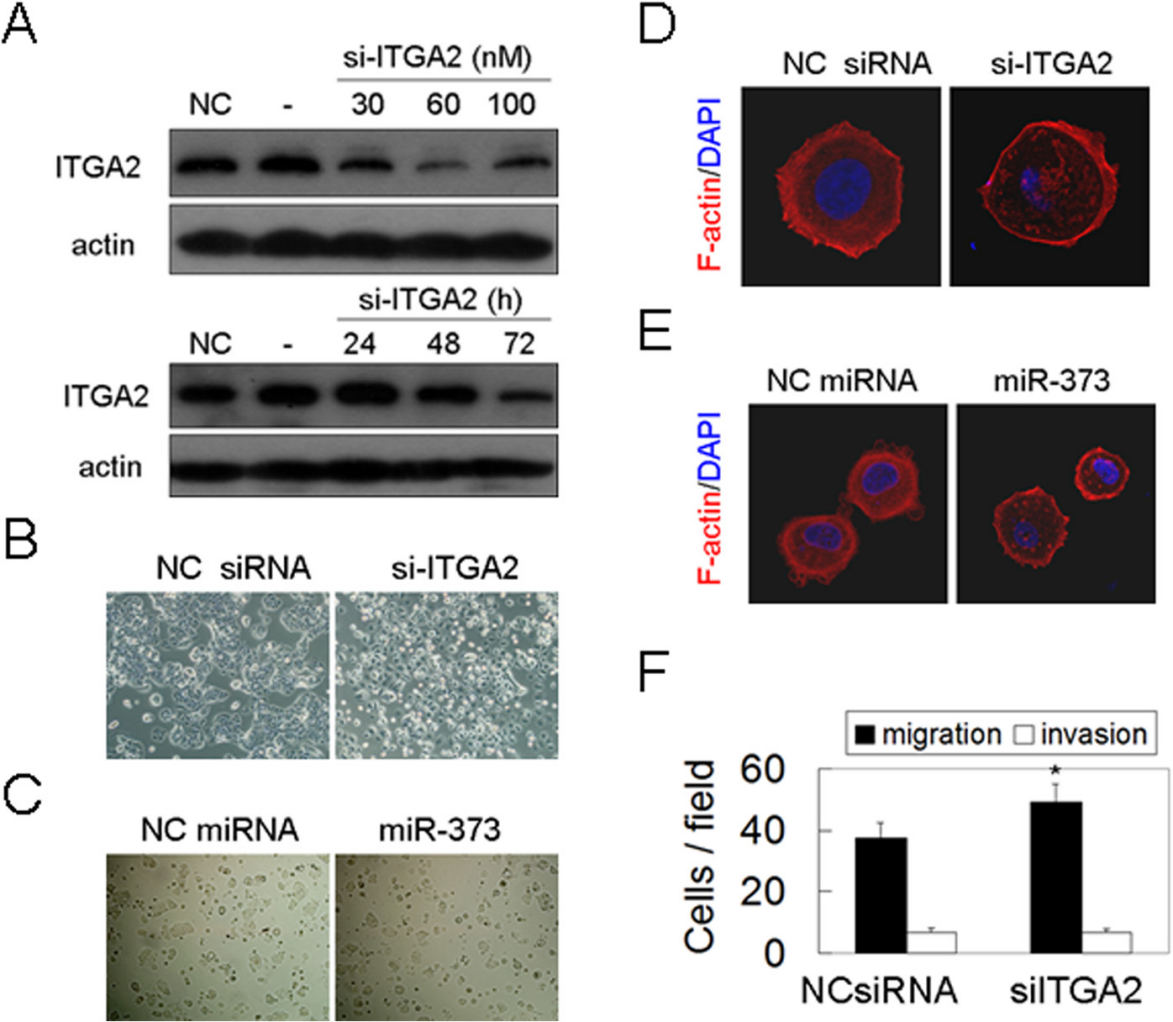
Silencing of ITGA2 detached cell-cell interactions and stimulated cancer cell migration. (A) MCF-7 cells were transfected with anti-ITGA2 siRNAs at the indicated concentration (upper panel) or at the indicated time (lower panel), and then ITGA2 protein level was determined by western blot. (B) MCF-7 cells were transfected with siRNAs as indicated, and then the cell morphology was analzyed. (C) MCF-7 were transfected with miRNA precursor as indicated, the cell morphology was then analzyed as described in (B). (D) Cells were treated as described in (B), and then F-actin was detected by immunofluorscent staining. (E) Cells were treated as described in (C), and then F-actin was analyzed as described in (D). (F) MCF-7 cells were transfected with siRNAs as indicated, the migration and invasion was analyzed by transwell assay (*p<0.05).

To investigate whether miR-373-downregulated ITGA2 plays an important role in invasion and metastasis, the ITGA2 level was knocked down by anti-ITGA2 siRNAs (Fig 4A). We found that silencing of ITGA2 damaged the cell-cell adhesion and detached cell-cell interaction (Fig 4B), similar to the effects of miR-373 overexpression (Fig 4C). And silencing of ITGA2 induced the deploymerization of stress fiber F-actin (Fig 4D), similar to the effects of miR-373 overexpression (Fig 4E). ITGA2 knockdown stimulated cancer cell migration but did not affect cancer invasion (Fig 4F), similar to our previous report that the influences of miR-373 on cancer migration [19]. Collectively, our data showed that down-regulation of ITGA2 by miR-373 induces cancer cell migration by detaching the cell-cell adhesion and regulating deploymerization of cytoskeleton.

### ITGA2 is an important target in miR-373-induced migration

**Fig 5:**
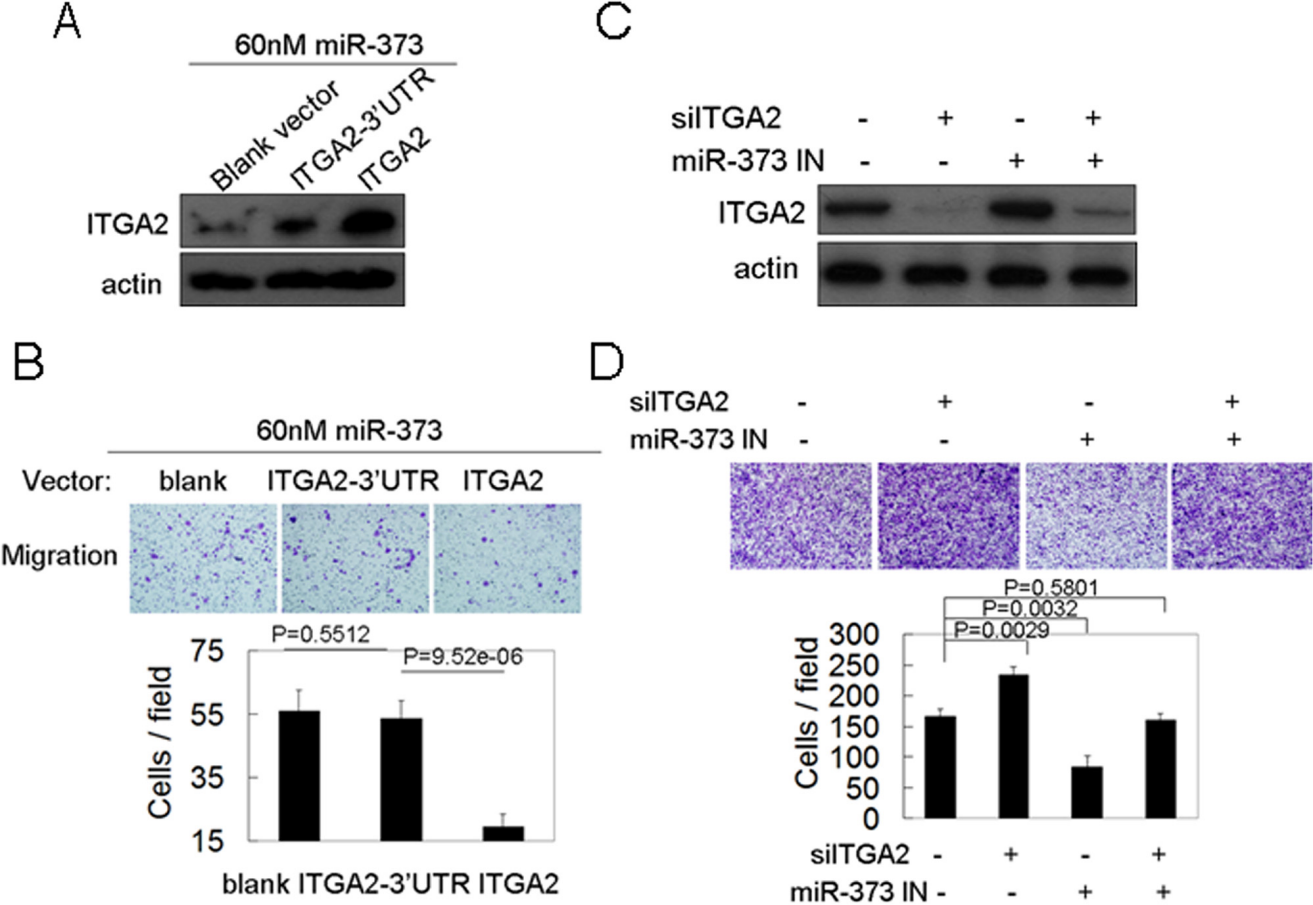
The co-expression of ITGA2, not ITGA2-3’UTR, could block miR-373-induced cancer cell migration. (A, B) MCF-7 cells were co-transfected by 60nM miR-373 precursor together with the indicated plasmids, and then ITGA2 protein level was analyzed by western blot (A), and the migration was analyzed by transwell assay (B). (C, D) MDA-MB-231 cells were co-transfected with 60nM miR-373 inhibitor and 60nM anti-ITGA2 siRNAs, and then ITGA2 protein level and migration was analyzed as (A, B).

To determine whether the effect of miR-373 on cancer migration is mediated mainly through ITGA2, miR-373 precursor was co-transfected into MCF-7 cells, together with a vector expressing ITGA2 lacking its 3’UTR (ITGA2), or a vector expressing ITGA2 containing its 3’UTR (ITGA2-3’UTR). The ITGA2 level was significantly increased in the ITGA2-transfected cells due to the absence of miR-373 binding sites, although miR-373 down-regulated the endogenous ITGA2 expression level (Fig 5A), whereas the ITGA2 level was not increased in ITGA2-3’UTR-transfected cells because the expression of the ITGA2-3’UTR was inhibited by co-transfected miR-373 (Fig 5A). Co-transfection of ITGA2 blocked miR-373-induced cancer cell migration, but ITGA2 3’UTR did not (Fig 5B). Furthermore, the metastatic cancer cells MDA-MB-231 were co-transfected with anti-miR-373 and siRNA-targeted ITGA2. We found that ITGA2 knockdown could rescue the inhibitory effects of the inhibition of miR-373 on breast cancer cell migration (Fig 5C and 5D). These observations showed that ITGA2 is an important target gene in miR-373-induced migration.

### ITGA2 protein level is inversely associated with miR-373 level in breast carcinomas

**Fig 6:**
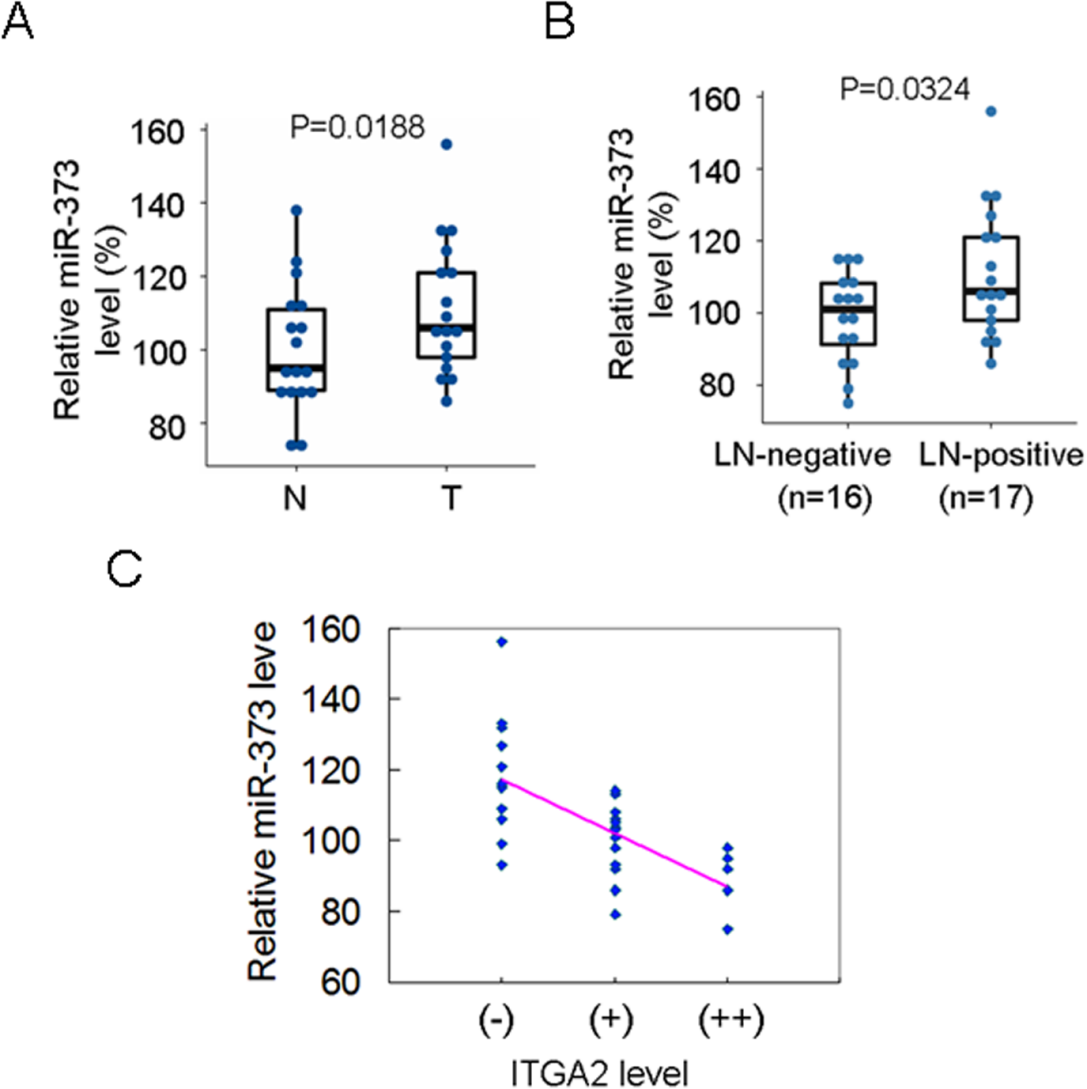
ITGA2 protein level was inversely associated with miR-373 level in clinical samples. (A) The miR-373 level between adjacent normal breast tissues (N) and breast cancers (T) was compared by qRT-PCR among 17 cases. The dotted lines represent the mean value. (B) The miR-373 level between LN-positive and LN-negative primary breast cancers was compared by qRT-PCR. (C) The correlation of miR-373 with ITGA2 was analyzed by a dot plot displaying assay. ITGA2 (-) indicates 0 points; (+), 3 points; (++), 4–5 points.

To further investigate the correlation of ITGA2 translation inhibition with miR-373 in breast carcinomas, the miR-373 level in primary breast cancers and matched adjacent non-cancerous breast tissues was further analyzed. Our data demonstrated that a higher miR-373 level was observed in primary breast samples, compared to adjacent non-cancerous breast tissues (P = 0.0188) (Fig 6A). The miR-373 level in LN-positive primary cancers was higher than that in LN-negative cancers (P = 0.0324) (Fig 6B). The Spearman’s rho correlation of miR-373 with ITGA2 protein level was analyzed based on the ITGA2 expression scores as previously described [20, 21]. A negative correlation was observed between miR-373 and ITGA2 in breast cancers (r = -0.663, P<0.001), and no patients showed higher ITGA2 level in breast cancer patients with higher miR-373 (Fig 6C). 11/15 (73.33%) of breast cancer patients with high miR-373 and low ITGA2 expression exhibited the LN-positive metastases. Altogether, these observations show that ITGA2 level was negatively associated with miR-373 level, miR-373-regulated ITGA2 translation inhibition is relevant to metastasis in breast cancer, and miR-373^high^/ITGA2^low^ may be as a prognosis biomarker for breast cancer patients.

## Discussion

The ITGA2 level was lost in primary cancers; however, the mechanism of ITGA2 loss is still unclear. Previous studies showed that phosphatase of regenerating liver-3 suppressed ITGA2 expression in ovarian cancer cells at the transcription level [22], and COX-2 increased ITGA2 expression through the EP1/PLC/PKCα/c-Src/NF-κB signal transduction pathway in human chondrosarcoma cells [23], the strikingly decreased methylation at key CpG sites within the promoter of ITGA2 gene in prostate tumors, compared with normal prostate tissue [24]. Here, we found that the reduction degree at protein level was more than that at mRNA level, suggesting that other regulatory mechanism participates into the loss of ITGA2 expression in primary cancers, in addition to the regulation at transcription level. We further indicate miR-373 directly decreases ITGA2 expression via translation inhibition but did not change the mRNA level of ITGA2. Therefore, we speculate that up-regulation of miR-373 further suppresses the protein synthesis of ITGA2 such that ITGA2 protein level is further significantly down-regulated. Thus, a novel mechanism of the reduction of ITGA2 protein level is elucidated in this study.

Cell adhesion and detachment onto other cells and cell-extracellular matrix plays a prominent role during embryonic development, wound healing and cancer invasion and metastasis. The regulation of cell-cell, cell-matrix interactions is mediated by various adhesion receptors on the cell surface, such as integrins [25]. The ITGA2, mainly together with the β1 integrin subunit, serves as the principal receptor for matrix molecules (collagen, laminin, fibronectin, etc.) and cell surface proteins, such as E-cadherin and vascular cell adhesion molecules [25, 26]. The loss of ITGA2 expression was helpful for malignant progression and metastasis. However, some studies demonstrated that ITGA2 up-regulation mediated selective metastasis to different organs such as the liver in melanoma [27] and bones in prostate cancer [28]. The controversial effects of ITGA2 on cancer metastasis might be explained by our observations. We here found that ITGA2 protein level was significantly reduced in breast cancers compared the adjacent normal breast tissues, and ITGA2 silencing induced cancer migration, suggesting that the reduction of ITGA2 in primary cancer aided cancer metastasis. However, the ITGA2 protein level was significantly recovered in lymph node metastases compared to primary cancer, suggesting that ITGA2 up-regulation in detached cancer cells in blood vessels or the lymphatic system was helpful for location and interaction with ECM in lymph node and distant tissues. Therefore, we speculate that ITGA2 may play a dual role in cancer metastasis. In the early process of cancer metastasis, ITGA2 reduction helps cancer cells to detach the primary cancer; in late phase cancer, ITGA2 recovery helps cancer cells to locate in lymph node and distant organs.

In addition, previous studies found that ITGA2 up-regulation in metastases of distant organs mediated selective metastasis to distant organs. However, our results demonstrated that ITGA2 protein level is markedly increased in lymph node metastases. The phenomenon showed that ITGA2 up-regulation was not requisite for selective metastasis for specific organs.

MiR-373 is an oncogene and can induce tumor initiation and progression in testicular germ-cell tumors by targeting tumor suppressor LATS2, and invasion and metastasis by targeting CD44, RECK, mTOR and SIRT1 in breast, colon cancer and fibrosarcoma, respectively [29–32]. Here, we showed that ITGA2 was a novel target gene of miR-373 in the regulation of migration. Our data reinforced the important role of miR-373 in the regulation of invasion and metastasis. In clinical samples, we further demonstrated that ITGA2 protein level was negatively associated with miR-373. Breast cancer patients with high miR-373 and low ITGA2 exhibited the LN-positive metastases.

In summary, the translation of ITGA2 mRNA was directly inhibited by miR-373. The miR-373-induced ITGA2 loss stimulates cancer migration. ITGA2 protein level was inversely associated with miR-373 level in breast carcinomas. Most of breast cancer patients with high miR-373 and low ITGA2 expression exhibited the lymph node-positive metastases. MiR-373^high^/ITGA2^low^ may be as a prognosis biomarker for breast cancer patients.

## Materials and Methods

### Cell culture, reagents and tissue samples

MDA-MB-231, MDA-MB-468 and MCF-7 breast cancer cells were obtained from American Type Culture Collection (ATCC, MD, USA) and cultured in L15 and DMEM medium with 10% FBS under standard conditions, respectively.

MiR-373 precursor, inhibitor and negative control (NC) miRNAs were purchased from Ambion (Huston, TX). Anti-ITGA2 siRNAs and NC siRNAs were purchased from Genepharma (Shanghai, China). Anti-ITGA2 siRNA: sense, 5′-ACUGGAGGUUUUCUCACAUTT-3′; antisense, 5′-AUGUGAGAAAACCUCCAGUTT-3′. NC siRNAs was designed as previously described [33]. The anti-ITGA2 antibodies for western blotting and IHC were from Santa Cruz (CA, USA) and Abcam (Cambridge, UK), respectively. The Hairpin-it-miRNAs qPCR quantitation kit was from GenePharma (Shanghai, China).

Clinical samples, including primary breast cancers, lymph node metastasis tissues, and adjacent non-tumoral breast specimens, were obtained at the time of surgery from 53 previously untreated breast cancer patients at Nanfang Hospital of Southern Medical University, from March 2012 to April 2013. Pathological diagnosis, as well as ER, PR, Her2, and Ki-67 status, was verified by two independent pathologists. The tissue specimen collection was approved by the Internal Review and Ethics Boards at The Third Affiliated Hospital of Guangzhou Medicine University, and the written informed consent was obtained from each patient.

### Immunohistochemistry (IHC) staining

IHC was performed according to standard LSAB protocol (Dako, Carpinteria, CA) using anti-ITGA2 antibody. IHC results were assessed by two independent pathologists who were blinded to the origination of the samples and subject outcome. The German semi-quantitative scoring system, considering the staining intensity and area extent, was applied as previously described with minor modification [21]. Briefly, each specimen was assigned a score according to the extent of stained cells (≤10% positive cells, 0; 11–50% positive cells, 2; 51–80% positive cells, 3; >80% positive cells, 4), and its intensity (no staining, 0; weak staining, 1; moderate staining, 2; strong staining, 3). Points for percentage of positive cells and staining intensity were added, and these samples were then attributed to four groups according to their overall score: negative expression (-), less than 10% of cells stained positive, regardless of intensity; weak expression (+), 3 points; moderate expression (++), 4–5 points; and strong expression (+++), 6–7 points.

### Oligonucleotide transfection

MiR-373 precursor or inhibitor, and anti-ITGA2 siRNAs were transfected with RNAiMAX. The luciferase reporter gene vector, ITGA2, ITGA2-3’UTR vector and DNA-RNA (miRNA precursor or inhibitor) mix were transfected with Lipofectamine 2000. Forty-eight hours after transfection, except from the indicated treatment time, cells were harvested for analysis in the next step.

### Migration and invasion assays

In vitro migration and invasion assays were performed using Transwell chambers as previously described [19]. Briefly, the cells were detached and resuspended in serum-free medium. 2×10^5^ cells were added in the upper Transwell chamber, and medium, supplemented with 10% fetal calf serum, was added to the bottom chamber. The filtered cells were stained with 5% crystal violet. For invasion assays, similar Transwell chambers coated with Matrigel were used to analyze the invasive ability.

### Western blotting

Cellular proteins were prepared according to the method previously described [34, 35]. In breif, the total proteins were separated by 10% SDS-PAGE. The proteins were detected with anti-ITGA2 and actin antibodies.

### Real-time qRT-PCR for miRNA and mRNA

Total RNA was extracted using Trizol total RNA isolation reagent (Invitrogen). The mature form of miR-373 was quantified using Hairpin-it-miRNAs qPCR quantitation kit. In brief, microRNAs were reversely transcribed by stem-loop RT primer, quantitative PCR was then carried out (n = 3). The U6 small nuclear RNA was used as an internal control. The mRNA level of ITGA2 was quantified by the specific primer sets (n = 3). GAPDH was used for normalization.

### Constructs

The ITGA2 3’UTR sequence was cloned into psiCHKECK-2 luciferase vectors (Promega). The ITGA2 cDNA sequence and ITGA2-3’UTR full sequence containing ITGA2 3’UTR sequence were cloned into pcDNA3.1/6His vectors (Invitrogen). The mutant luciferase reporter constructs of ITGA2 3’UTR were generated using a QuikChange Site-Directed Mutagenesis Kit (Stratagene).

### Luciferase reporter assay

The luciferase reporter constructs and their mutation constructs were co-transfected into breast cells together with miR-373 precursor or miR-373 inhibitor, respectively. Luciferase activity was measured 48 hours after transfection using the dual-luciferase reporter assay system (Promega). Firefly luciferase activity was used to normalize Renilla luciferase activity for each transfected well (n = 3).

### Immunofluorescence staining

MCF-7 cells were transfected with the 30 nM miR-373 precursor or 60 nM anti-ITGA2 siRNAs, respectively. The transfected cells were then detached and re-cultured on glass cover slips. Cells were then fixed with 4% paraformaldehyde, permeabilized with 0.1% Triton X-100, and cultured with Rhodamine phalloidin for staining F-actin. Cellular nuclei were stained with DAPI. Staining for F-actin and DAPI was visualized and captured using a confocal laser microscope (Nikon, Japan).

### Statistical analysis

All statistical analyses were performed by the SPSS (V16.0) program. The correlations between ITGA2 expression and clinicopathological status were analyzed by using chi-square test (x^2^ test). The two-tailed independent Student’s t-test and Mann Whitney U test were used for comparisons between two groups. Spearman’s rho correlation test was applied to determine the correlations. The data are shown as the mean ± SD, except where stated otherwise.
